# Professional satisfaction of nurses in psychosocial rehabilitation services

**DOI:** 10.1192/j.eurpsy.2021.1233

**Published:** 2021-08-13

**Authors:** A. Zartaloudi, L. Karvouni, T. Adamakidou, M. Mantzorou

**Affiliations:** 1 Nursing, University of West Attica, Athens, Greece; 2 Nursing, Psychiatric Hospital of Athens “Dafni”, Athens, Greece

**Keywords:** Psychosocial rehabilitation, mental health nurses, job satisfaction

## Abstract

**Introduction:**

The increase of job satisfaction in mental health nurses, working in community mental facilities, helps them to become more efficient and understand the needs of individuals suffering from mental health problems.

**Objectives:**

To investigate sociodemographic and job characteristics, as well as the level of professional satisfaction of nurses working in psychosocial rehabilitation facilities of the psychiatric hospital of Athens, named Dafni.

**Methods:**

220 nurses, working in the field of psychosocial rehabilitation completed (a) a sociodemographic questionnaire, (b) Spectοr’s Job Satisfaction Survey (JSS).

**Results:**

Nursing staff consists of mental health nurses (44.1%) and nursing assistants (55.9%) in the present study. 90% of the participants were female; while the 65% were married, the 19.5% had a university-level education and the 25% had administrative responsibilities. The 44.1% of our sample worked in hostels, 41.8% in nursing homes and 6.8% in Community Mental Health Centers. More specifically, moderate levels of total professional satisfaction were observed. Concerning the dimensions of satisfaction, low satisfaction rates were recorded in “salary”, “promotion” and in “privileges and benefits”. High satisfaction rates were recorded in “supervision by their superiors”, “cooperation between colleagues” and “the nature of their work”, while moderate satisfaction rates were reported in “communication” within facilities, as far as the explanation of tasks and objectives is concerned.

**Conclusions:**

The results can be exploited by those with administrative and scientific responsibilities in the field of mental health in order to recognize nurses’ difficulties and solve their problems in psychosocial rehabilitation facilities.

